# Left Ventricular Remodeling Patterns in Primary Healthcare

**DOI:** 10.36660/abc.20180258

**Published:** 2020-01

**Authors:** Roberto de Castro Meirelles de Almeida, Antonio José Lagoeiro Jorge, Maria Luiza Garcia Rosa, Adson Renato Leite, Dayse Mary S. Correia, Evandro Tinoco Mesquita, Sergio Chermont, Jocemir Ronaldo Lugon, Wolney de Andrade Martins

**Affiliations:** 1 Curso de Pós-Graduação em Ciências Cardiovasculares da Universidade Federal Fluminense (UFF), Niterói, RJ - Brazil; 2 Curso de Pós-Graduação em Ciências Médicas da Universidade Federal Fluminense (UFF), Niterói, RJ - Brazil

**Keywords:** Cardiovascular Diseases/physiopathology, Ventricular Remodeling, Hypertrophy, Left Ventricular, Heart failure, Renal Insufficiency, Risk Factors/complications, Comorbidity

## Abstract

**Background:**

Left ventricular remodeling (LVR) is related to both non-fatal and fatal outcomes.

**Objective:**

To describe the geometric patterns of the LV and their associations.

**Methods:**

A total of 636 individuals between the ages of 45 and 99 years in Rio de Janeiro, Brazil, were submitted to clinical evaluation, laboratory tests, electrocardiogram, and tissue Doppler echocardiography (TDE). The difference between categories was tested with Kruskall-Wallis with post hoc tests, once all variables studied are non-normally distributed and Pearson’s Qui-square (categorical variables). Gross and adjusted ORs were estimated by logistic regression. The level of significance was 5% for all tests. Subjects had LVR characterized as: normal geometry (NG), concentric remodeling (CR), concentric hypertrophy (CH), and eccentric hypertrophy (EH).

**Results:**

The prevalence of altered patterns was 33%. Subjects presented NG (n = 423; 67%); EH (n = 186; 29%); CH (n = 14; 2%); and CR (n = 13; 2%). The variables of gender, age, level of education and albumin/creatinine ratio (A/C), showed a relationship with the chance of EH even after adjustment.

**Conclusion:**

Approximately one third of the studied individuals had LVR and were at risk for developing heart failure. Altered A/C in urine was associated with EH, indicating an early relationship between cardiac and renal dysfunction.

## Introduction

Ventricular remodeling is a continuous process of responses to the various injuries to the myocardium. Changes in left ventricular (LV) geometry, in its various patterns, are related to the incidence of non-fatal cardiovascular outcomes and long-term mortality, which are well-known markers of poor prognosis in various cardiovascular and systemic diseases.^1-6^ Changes in ventricular geometry are considered target organ lesions on the heart and make individuals with these lesions classified as being in stage B of heart failure (HF) as it is proposed by the American College of Cardiology Foundation and American Heart Association (ACCF/AHA).^7^

The pathophysiological mechanisms of ventricular remodeling vary according to the determining etiology. The diseases lead to pressure overload with increased systolic wall stress, gene activation, or direct myocardial injury followed by cell proliferation, fibrosis, collagen deposition, apoptosis, and remodeling of the ventricular geometry. The conditions that occur with volume overload lead to increased diastolic wall stress with linear stretching of cardiomyocytes, proliferation in parallel, and increased cavity diameters.^8^

Epidemiological data on the prevalence and incidence of changes in ventricular geometry in population seen in primary care are scarce and knowledge of different remodeling patterns may assist in the implementation of strategies for risk stratification in this population. The aim of this study was to describe the geometric patterns of the LV in the population aged ≥ 45 years assisted in primary care, and to examine the association between ventricular remodeling and demographic and clinical variables.

## Methods

This study is part of the *Digitalis trial* that aimed to determine the prevalence of HF in the population studied.^9^

### Procedures for random sample selection and patient inclusion

We selected 26 primary care units in the city of Niterói, Rio de Janeiro, Brazil, between July 2011 and December 2012. The selection of units was done by a computer-generated random sequence program, in which the weight of each unit was proportional to the number of individuals assisted. In each unit, 50 subjects were randomly selected, including 30 individuals for participation and 20 for replacement in case of negative response. The selected total population was 1050. Nine hundred forty-two individuals confirmed the presence and 666 individuals attended the scheduled visit. Inclusion criteria were age between 45 and 99 years old and informed consent. Five individuals who did not complete the questionnaire were excluded, 6 did not perform the tissue Doppler echocardiography (TDE), and 20 did not perform the measurement of B-type natriuretic peptide (BNP). At the end of the study, 636 patients completed the necessary requirements: structured questionnaire, physical examination, anthropometric data, BNP, electrocardiogram (ECG) at rest and TDE.

### Definitions

All individuals selected for the study were subjected to an assessment carried out in a single day and consisting of the following elements: clinical evaluation, laboratory tests, including BNP levels, ECG, and TDE.

TDE tests were performed according to the recommendations for the quantification of chambers of the *American Society of Echocardiography* and the *European Association of Echocardiography*. (LANG, 2015). Indexing was performed by body surface area. The left ventricular mass (LVM) was estimated by Devereux et al. (DEVEREUX, 1986) and relative wall thickness (RWT) by the formula where RWT is equal to twice the diastolic posterior wall divided by the diameter of the LV. RWT values ≥ 0.42 and indexed LV ≥ 115 g/m^2^ for men and ≥ 95 g/m^2^ for women were considered abnormal. The subjects were grouped into four remodeling models: normal geometry, concentric remodeling, concentric hypertrophy, and eccentric hypertrophy, according to the Guidelines of the American Society of Echocardiography.^10^

Patients were classified in stages of chronic kidney disease (CKD) according to estimated glomerular filtration rate (eGFR) calculated by KDIGO formula (Kidney Disease: Improving Global Outcomes). Stage 1: eGFR >90 mL/min; stage 2: eGFR 60-89 mL/min; stage 3: eGFR 30-59 mL/min; stage 4: eGFR 15-29 mL/min; and stage 5: eGFR <15 mL/min.

Individuals with BMI ≥ 30 kg / m2 were considered obese. Diabetic patients were defined by previous history of diabetes. The study classified as hypertensive individuals those who reported being hypertensive, were on medication to treat hypertension or had a mean systolic blood pressure (SBP) ≥ 140 mmHg or mean diastolic blood pressure (DBP) ≥ 90 mmHg.

### Statistical analysis

Statistical analysis was performed using SPSS v 21.0 (Chicago, Illinois, USA). Continuous variables were expressed as median and interquartile range (50 %(25-75%)). Categorical variables were expressed in absolute numbers and/or percentages. For comparison between groups, the qui-square test was employed. All continuous variables were tested for normality with the Shapiro-Wilk test with pos-hoc test and for all of them the Ho (null hypothesis) of equality was rejected, that is, none of them had normal distribution. To that extent, the difference between those variables and the phenotypes was tested with the Kruskall-Wallis test. We estimated crude and adjusted odds ratios by logistic regression. In all comparisons, bilateral tests were utilized, and p values < 5% were considered statistically significant.

### Ethical considerations

This study was conducted in accordance with the principles set out in the Declaration of Helsinki revised in 2000 (Scotland, 2000). The study was previously approved by the Universidade Federal Fluminense under n° CAAE: 0077.0.258.000-10, and informed written consent was provided by all participating patients.

## Results

The study evaluated 636 individuals of 59.5 ± 10.3 years old (62% women, 63% non-whites). The subjects were classified according to the geometry of the LV: normal geometry in 423 (67%); eccentric hypertrophy in 186 (29%); concentric hypertrophy in 14 (2%); and concentric remodeling in 13 (2%). Demographic data of the subjects are listed in Table 1. The variables of age, gender, level of education, high blood pressure, pulse pressure, albumin/creatinine ratio, and the sodium/creatinine ratio in urine were statistically significant between the remodeling patterns. Hypertension and diabetes mellitus were the most prevalent comorbidities in patients with concentric hypertrophy, while coronary artery disease and obesity occurred more frequently in the group with concentric remodeling (Table 1). Table 2 lists the main echocardiographic changes.

**Table 1:** Demographic and clinical characteristics of the selected individuals characterized by left ventricular remodeling patterns

Variables	Normal geometry n = 423	Eccentric hypertrophy n = 186	Concentric hypertrophy n = 14	Concentric remodeling n = 13	p-value (*)
Age (years)	56 (50-64)	58 (47-70)	63 (55-71)	61 (53-71)	< 0.0001
Female (%)	57.4	71.5	78.6	53.8	0.005
Non-white (%)	63.5	63	57	61.5	0.812
SBP (mmHg)	133.7 (121-147)	133(115.5-134.9)	144.5 (126-175)	135.6 (120.2-156)	0.148
DBP (mmHg)	82.3 (74.3-90.0)	76 (73.5-91)	83.5 (75.9-91.5)	80 (72-90)	0.385
PP (mmHg)	517 (42-60)	51 (36-59)	60 (47-59)	55 (46-67)	0.004
HR (bpm)	70 (63-78)	72 (65-80)	74 (64-83)	69 (61-77)	0.468
BMI (kg/m^2^)	27.2 (24,5-30.6)	28.6 (19.4-35.8)	26.8 (23.4-30.6)	27.7 (24.3-30.7)	0.881
Glycemia (mg/dL)	101 (92-113)	107 (92-125)	104 (92-125)	100 (92-115)	0.734
GFR (mL/min)	83.4 (71.7-96.1)	84.1 (74.6-92.8)	67.7 (53.6-85.0)	82.3 (67.2-94.1)	0.021
Uric acid (mg/dL)	5.2 (4.3-6.4)	4.5 (3.8-5.9)	4.6 (3.9-5.9)	5.0 (4.1-6.1)	0.266
Cholesterol (mg/dL)	214 (188-243)	201 (157-221)	208 (184-250)	215 (185-245)	0.507
Alb/Creat (mg/g)	9.3 (5.5-18.7)	8.0 (5.2-170.1)	16.2 (9.9-42.9)	11.8 (6.2-38.9)	0.018
sod/creat in urine	120.9 (77-160)	108.3 (48-200)	145.4 (77-235)	126.2 (89-185)	0,205
BNP (pg/mL)	14 (10-25)	17 (11-41)	35 (14-120)	21 (11-42)	< 0.0001
Albuminuria (mg)	10.5 (5.4-20.8)	8.5 (4.4-65.4)	13.7 (5.8-30.8)	12.8 (7-34.5)	0.049
Hypertension (%)	54.6	63.4	71.4	53.8	0.150
Diabetes (%)	24.3	23.7	50.0	30.8	0.159
CAD (%)	8.7	7.5	14.3	15.4	0.657
Obesity (%)	28.7	32.3	28.6	46.2	0.488

SBP: systolic blood pressure; DBP: diastolic blood pressure; PP: pulse pressure; HR: heart rate; BMI: body mass index; GFR: estimated glomerular filtration rate; Alb/Creat: albumin/creatinine ratio; sod/creat: sodium/creatinine ratio; BNP: type B natriuretic peptide; CAD: coronary artery disease;

(*) p = difference between the patterns of remodeling performed with the Kruskall-Wallis test 1-way ANOVA for multiple comparisons (all pairs) and Pearson's chi-squared test for differences of proportion.

**Table 2:** Echocardiographic parameters of the selected individuals characterized by left ventricular remodeling patterns

Variables	Normal geometry n = 423	Eccentric hypertrophy n = 186	Concentric hypertrophy n = 14	Concentric remodeling n = 13	p-value (*)
LVEF (%)	61 (58-65)	63 (58-64)	59 (50-64)	60 (56-63)	0.01
LVMi (g/m^2^)	82.8 (72.2-92.0)	84 (70.7-93.4)	120 (105.6-150.71)	116.3 (102.4-127.3)	< 0.0001
LAVi (ml/m^2^)	20.8 (17.1-24.5)	18 (14-22.3)	23.6 (19.2-33.1)	22.6 (19.2-27.5)	< 0.0001
EDVi (ml/m^2^)	60.1 (51.8-67.2)	41.7 (36.4-49.4)	60.5 (52.1-75.9)	74.7 (67.7-83.2)	< 0.0001
E'(cm/s)	10(8-12)	12(7.5-13)	7.7(6-10.6)	9(7-11)	< 0.0001
E (cm/s)	64 (54-76)	70(52-93)	68(51-75)	63 (52-78)	0.532
A (cm/s)	65 (53-81)	76(59-99)	69(56-98)	71 (57-86)	0.242
E/A ratio	1.0 (0.7-1.3)	1.1 (0.6-1.3)	0.9 (0.7-1.1)	0.8 (0.7-1.2)	0.003
E/E' ratio	6.4 (5.3-7.7)	7.1 (4.7-8.7)	8.0 (5.7-10.0)	7.1 (5.7-8.4)	0.008
SWT (mm)	8(7-8)	9(8-10)	10(10-12)	9(8-9)	< 0.0001
LVDD (mm)	47 (44-50)	40 (34.5-41.5)	43.5 (41.7-47.8)	52 (49-54,2)	< 0.0001
PWT (mm)	8(7-8)	9(8-10)	10(10-12)	8(8-9)	< 0.0001

LVEF: left ventricular ejection fraction; LVMi: indexed left ventricular mass; LAVi: indexed left atrial volume; EDVi: indexed end-diastolic volume; E': mitral annular early diastolic velocity; E: early mitral inflow velocity; A: peak mitral inflow velocity at atrial contraction ; LVDD: LV diastolic diameter; SWT: septal wall thickness; PWT: posterior wall thickness

(*) p value - the difference between the patterns of remodeling performed with Kruskall-Wallis test 1-way ANOVA for multiple comparisons (all pairs).

Table 3 presents the crude and adjusted *odds ratio* of eccentric hypertrophy versus normal geometry, the only remodeling pattern with sufficient prevalence to achieve adequate power for conducting a multiple analysis. The variables of gender, age, level of education and albumin/creatinine ratio showed a relationship with the risk of eccentric hypertrophy even after adjustment.

**Table 3:** Crude and adjusted odds ratios of eccentric hypertrophy versus normal geometry

Variables	OR (IC 95%)	ORa (IC 95%)
**Gender**		
Female	1.80 (1.25-2.60)	1.75 (1.17-2.61)
Male	1	1
**Age range**		
70-99 years	2.33 (1.54-3.52)	2.04 (1.28-3.26)
45-69 years	1	1
**Skin color**		
Black	1.20 (0.812-1.774)	
Non-black	1	
**Level of education**		
≤ Elementary school	1.86 (1.32-2.62)	1.59 (1.07-2.34)
> Elementary School	1	1
**Per capita income**		
≤ 1 MW	1.52 (1.00-2.30)	
> 1 MW	1	
**Smoking in life**		
Smoker or ex-smoker	0.91 (0.65-1.28)	
Never smoked	1	
**Alcohol consumption - risk drinker**		
Yes	0.57 (0.29-1.10)	
No	1	
**Physical activity**		
Sedentary and irregular	0.82 (0.57-1.92)	
Active or very active	1	
**Obesity**		
Yes (BMI ≥ 30 kg/m^2^)	1.16 (0.81-1.68)	
No (BMI < 30 kg/m^2^)	1	
**Hypertension**		
Yes	1.69 (1.13-2.52)	1.23 (0.79-1.92)
No	1	1
**Type 2 diabetes**		
Yes	0.94 (0.63-1.41)	
No	1	
**Uric acid altered**		
Yes	1.04 (0.68-1.59)	
No	1	
**Coronary artery disease**		
Yes	0.78 (0.41-1.47)	
No	1	
**Albumin/creatinine ratio**		
≥ 30 mg/g	1.82 (1.19-2.76)	1.64 (1.05-2.57)
< 30 mg/g	1	1
**Sodium/creatinine ratio in urine**		
> 10.39 g/dL	1.64 (1.08-2.47)	1.34 (0.86-2.09)
≤ 10.39 g/dL	1	1
**Chronic kidney disease (CKD)**		
Stages 3,4,5	1.43 (0.84-2.43)	
Stages 1,2	1	

OR: odds ratio; Ora: odds ratio adjusted; CI: confidence interval; MW: minimum wage; BMI: body mass índex; Uric acid altered: male > 7.0 mg/dL and female > 6.0 mg/dL; CKD: chronic kidney disease estimated by glomerular filtration rate (KDIGO) stages 1, 2, 3, 4, and 5

**Figure 1:**
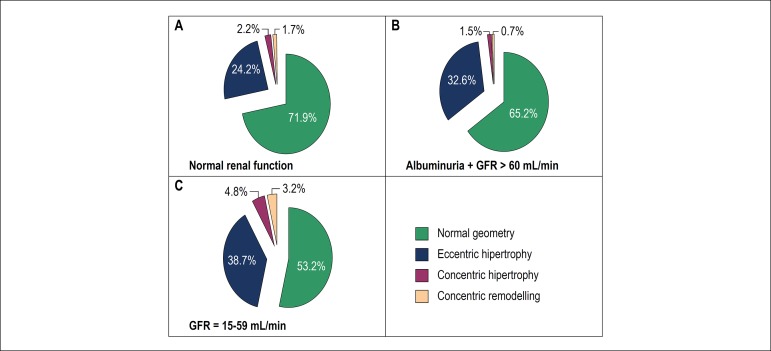
A, B, and C: Evaluation of parameters of renal function in different patterns of left ventricular remodeling.

Figure 1 shows the distribution of the patterns of left ventricular remodeling in patients without changes in renal function (A); in patients with subclinical changes demonstrated by microalbuminuria (B); and in those with established kidney disease (C).

## Discussion

In the present study, with individuals assisted in primary care aged 45 years or more, the main pattern of ventricular geometric changes was eccentric LVH. The change was more prevalent in women, older patients, patients with lower educational levels, and patients with hypertension and renal dysfunction.

The differences observed in this study compared to other studies in Europe and the United States can be explained by reasons similar to those reported in the study by Schvartzman et al.^3^ The stature of Brazilians is lower than that of Europeans and North American Caucasians, impacting the LV mass indexed by the body surface.

Ventricular remodeling throughout life occurs as an adaptive response to aging, exposure to risk factors for cardiovascular disease and myocardial injury.^11^ A study carried out in the community with 4492 participants (mean age 51 years and 59% women) showed that 64% had normal geometry, 18% had concentric remodeling, 13% had eccentric hypertrophy, and 5% had concentric hypertrophy. Our data are similar to the population with normal geometry (64 vs. 67%), but different in relation to remodeling standards, especially regarding concentric remodeling (18% vs. 2%) and eccentric hypertrophy (13% vs. 29%). These differences can also be explained by the greater number of hypertensive and diabetic patients observed in our study in relation to the trial of Lieb et al.^11^

A study conducted in the community by Teh et al.^12^ assessed the prevalence of the four remodeling models in 108 patients aged >70 years, in which 56% were women, 84% had hypertension, and 20% had diabetes.^12^ Although the data of Teh et al.^12^ were obtained in an older population, they are similar to ours in relation to the higher prevalence of eccentric hypertrophy observed in the sample (26% vs 29%), showing that there seems to be an increased prevalence of eccentric remodeling in older individuals and those with more comorbidities.

Aging is directly related to the progression of cardiac remodeling, most likely due to exposure to multiple cardiovascular risk factors. This finding was present in our study as well as in the literature.^13^

We observed an association between female gender and the presence of LV eccentric hypertrophy after adjusting for other variables (OR, 95% CI, 1.75 [1.17 to 2.61]). There are differences in cardiac structure and function in relation to gender, and these differences appear to be more pronounced in the presence of risk factors for HF with preserved ejection fraction (HFpEF), and they can be explained by sexual dimorphism.^14^ A study evaluating changes in LV stiffness in 1,402 individuals in the community observed an increase in stiffness with aging, which is increased in women more frequently than in men.^15^ A study involving 318 healthy adults from the Framingham Heart Study who underwent MRI to determine the reference values of LV parameters observed a greater increase in the linear dimensions of the LV after adjustment for body surface area in women than in men (p < 0.001).^16^

Our data showed that low educational level had an association with eccentric hypertrophy. Such a result can be explained by greater exposure to risk factors, less understanding about self-care, and less adherence to drug treatment.

Microalbuminuria is an important cardiovascular risk marker,^17^ and the data presented here showed that individuals with changes in ventricular geometry had high levels of urinary albumin and worsening renal function assessed by GFR. Individuals with impaired renal function have a progressive increase in eccentric hypertrophy, which may reflect heart disease with concomitant loss of kidney function. Both concentric and eccentric patterns reflect hypertensive nephropathy, which progresses with structural heart disease. Studies show the existence of an association between albuminuria, remodeling, and cardiovascular disease. Increased urinary albumin excretion is associated with changes in ventricular remodeling in patients with hypertension. Patients with hypertension who have albuminuria regression disability have a higher incidence of cardiovascular disease.^18^ Our data show there is a strong association between the albumin/creatinine ratio and the development of eccentric LV hypertrophy.

This study was the first in the Brazilian primary care population to specifically study the LV geometry with inclusion of RWT.

The greater inclusion of female patients is noteworthy as a limitation to the study. This was due to greater adherence of women to the study protocol. Similarly, the greater adherence to therapy may have influenced the remodeling patterns in females, and such adherence was not measured.

## Conclusion

One third of individuals attending primary care between the ages of 45 and 99 years, in the sample analyzed, had LVR and were at risk for developing HF. Altered albumin/creatinine ratios in urine was associated with eccentric hypertrophy, indicating an early relations hip between cardiac and renal dysfunction.
